# Evidence Gaps in Total Hip Arthroplasty: A Registry-Based Study of Trial Discontinuation and Nonpublication From 2000 to 2024

**DOI:** 10.7759/cureus.117850

**Published:** 2026-10-13

**Authors:** Carson R Sautter, Daxton J Kennington, Mark Gamadia, Alexis Ortega, Connor Parry, Keith Kotecki

**Affiliations:** 1 College of Osteopathic Medicine, Des Moines University, Des Moines, USA; 2 College of Osteopathic Medicine, Kansas City University, Kansas City, USA; 3 Department of Orthopedic Surgery, Valley Hospital Medical Center, Las Vegas, USA

**Keywords:** adult reconstruction, hip replacement, orthopedic surgery, research transparency, total hip arthroplasty

## Abstract

Background

Clinical trials play a vital role in advancing evidence-based orthopedic care, yet trial discontinuation and nonpublication limit transparency and reduce the availability of clinically valuable data. In total hip arthroplasty (THA), incomplete dissemination of trial outcomes may have important ethical and clinical implications.

Methods

A registry-based cross-sectional analysis of randomized Phase III and IV THA clinical trials registered on ClinicalTrials.gov between 2000 and 2024 was performed. Trials registered at least two years before the search date (January 2026) were included. Trial characteristics, including funding source, intervention type, enrollment size, blinding, number of centers, and recruitment location, were extracted. Publication status was determined through registry-linked publications and independent database searches. Associations were analyzed using chi-square or Fisher’s exact tests (p < 0.05).

Results

Of 168 identified trials, seven unrelated to THA were excluded, leaving 161 trials for analysis. Fifty trials (31.1%) were discontinued, and 111 (68.9%) were completed. Among completed trials, 26 (23.4%) remained unpublished. Discontinuation was significantly associated with intervention type (p = 0.0015) and blinding (p = 0.022), with higher rates in device trials (59.3%) and unblinded studies (55.9%). Nonpublication was significantly associated with industry funding (39.3% vs. 18.1%, p = 0.022) and intervention type (p = 0.044).

Conclusions

A substantial proportion of THA clinical trials are discontinued or remain unpublished, contributing to data loss and potential bias in the orthopedic literature. Improved reporting compliance is needed to support transparency and evidence-based orthopedic practice.

## Introduction

Clinical trials are essential to advancing surgical innovation and evidence-based practice, providing the foundation for evaluating the safety and effectiveness of new techniques, implants, and perioperative strategies [1]. Total hip arthroplasty (THA) is one of the most commonly performed orthopedic procedures worldwide, with procedural volume continuing to increase because of an aging population, expanding surgical indications, and advances in implant technology. Recent projections estimate that primary THA utilization in the U.S. will continue to rise substantially over the coming decades, underscoring the need for high-quality clinical evidence to guide implant selection, surgical techniques, and perioperative management. In THA, transparent dissemination of trial results is critical to informing clinical guidelines, guiding device development, and improving patient care [2]. Despite ongoing efforts to promote transparency, a substantial proportion of clinical trials across medical disciplines remain discontinued or unpublished, with estimates suggesting that 30-50% of studies never disseminate their findings [3-5].

Failure to report trial results raises significant ethical and scientific concerns. Participants assume risks with the expectation that their involvement will contribute to generalizable medical knowledge; this obligation is undermined when studies are discontinued without reporting or completed trials remain unpublished [1]. Nonpublication contributes to research inefficiency, unnecessary duplication of studies, financial waste, and potential delays in recognizing safety concerns or ineffective interventions [5,6].

These challenges are particularly relevant in orthopedic surgery, where THA is among the most frequently performed procedures worldwide and represents a substantial clinical and economic investment [7]. Although U.S. legislation, including the Food and Drug Administration Amendments Act, mandates trial registration and results reporting, compliance remains inconsistent [2]. The primary objective of this study was to evaluate the prevalence of trial discontinuation and nonpublication among randomized Phase III and Phase IV THA clinical trials registered on ClinicalTrials.gov. The secondary objective was to identify trial characteristics associated with trial discontinuation and nonpublication.

## Materials and methods

A comprehensive search of ClinicalTrials.gov, maintained by the U.S. National Library of Medicine, was performed on January 1, 2026, using the keyword “hip arthroplasty” to identify eligible randomized Phase III and Phase IV interventional clinical trials evaluating THA. Studies registered between January 1, 2000, and January 1, 2024, were eligible for inclusion. To allow adequate time for trial completion and publication, only studies registered at least two years before the search date were included. Studies were excluded if they were unrelated to THA, employed nonrandomized designs, involved Phase I or II investigations, enrolled pediatric populations, were ongoing after January 1, 2024, or lacked a clearly defined intervention.

Key trial characteristics, including recruitment status, funding source, enrollment size, intervention type, blinding methodology, number of study centers, and geographic recruitment location, were abstracted from ClinicalTrials.gov. Search results were independently screened by two reviewers using the predefined inclusion and exclusion criteria, with discrepancies regarding study eligibility resolved through discussion until consensus was reached. Recruitment status was classified as completed, terminated, withdrawn, or suspended.

Publication status was determined by reviewing registry-linked publications on ClinicalTrials.gov and performing independent literature searches in PubMed, Embase, and Google Scholar using trial registration numbers, investigator names, and study titles. A publication was considered valid only if the reported outcomes corresponded to the registered trial objectives and intervention. Registry-posted results, conference abstracts, preprints, dissertations, and theses were not considered evidence of publication. Publication searches were conducted independently by two reviewers and repeated to confirm nonpublication when applicable.

For trials in which no associated publication was identified, attempts were made to contact the principal investigators using publicly available contact information. A standardized email was sent, with up to two follow-up attempts at one-week intervals. Investigators were deemed unreachable if no response was received after these attempts. All investigators in the present study met the criteria for being unreachable.

The primary study outcomes were trial completion status (completed vs. discontinued, with discontinuation defined as terminated, withdrawn, or suspended) and publication status (published vs. unpublished among completed trials). Relationships between trial characteristics and outcomes were examined using contingency table analyses. Statistical significance was defined as p < 0.05. Chi-square testing was applied for categorical comparisons, with Fisher’s exact test or the Fisher-Freeman-Halton test used when expected cell counts were small. Effect sizes with 95% CIs were reported for applicable analyses. All statistical analyses were performed using R version 4.3.2 (R Foundation for Statistical Computing, Vienna, Austria).

## Results

Our initial search identified 168 registered hip arthroplasty clinical trials from 2000 to 2024. Seven trials were excluded during screening because they were unrelated to THA, leaving 161 trials that met the inclusion and exclusion criteria for the final analysis (Figure 1). These trials collectively enrolled approximately 43,387 participants across 32 countries, with the U.S. accounting for 44 trials (27.3%). Among international studies, Denmark accounted for 20 trials (12.4%), Canada for 15 (9.3%), and China for six (3.7%), representing the most frequent sites of trial activity.

**Figure 1:**
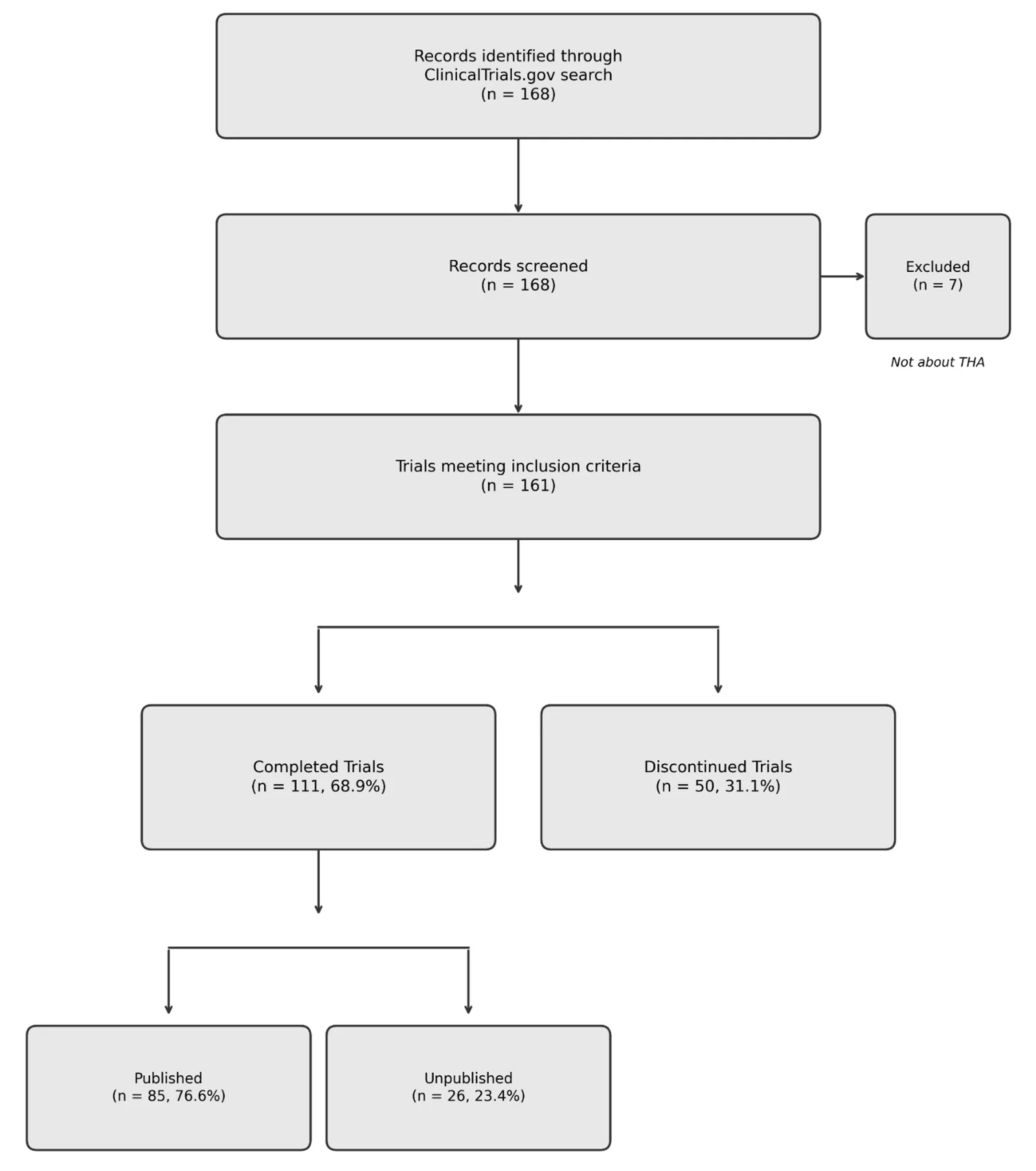
Search strategy for determining included randomized controlled trials. THA, total hip arthroplasty

Of the 161 registered hip arthroplasty clinical trials analyzed, 111 (68.9%) were completed, and 50 (31.1%) were discontinued (Figure 1, Table 1). A total of 43,387 participants were enrolled across all included trials (mean = 269 participants; range, 0-5,407), with 37,266 (85.9%) enrolled in completed trials and 6,121 (14.1%) in discontinued trials. Discontinuation was significantly associated with intervention type (p = 0.0015) and blinding method (p = 0.022). Device trials had a higher discontinuation rate than drug trials (59.3% vs. 23.6%), and unblinded trials were discontinued more frequently than quadruple-blinded trials (55.9% vs. 25.0%). No statistically significant associations were observed between trial completion status and funding source (industry vs. non-industry, p = 0.18), number of centers (p = 0.66), enrollment size (p = 0.30), or recruitment location (p = 1.0).

**Table 1:** Hip arthroplasty clinical trials by completion status.

Characteristic	Discontinued (n = 50)	Completed (n = 111)	Total (161)	Discontinuation rate	95% CI	p-value
Funding source						
Non-industry	32	83	115	27.8%	20.5-36.6%	0.18
Industry	18	28	46	39.1%	26.4-53.5%	
Intervention type						
Behavioral	0	1	1	0.0%	0.0-79.3%	0.0015
Device	16	11	27	59.3%	40.7-75.5%	
Dietary supplement	0	1	1	0.0%	0.0-79.3%	
Drug	29	94	123	23.6%	16.9-31.8%	
Procedure	7	14	21	33.3%	17.2-54.6%	
Other	0	8	8	0.0%	0.0-32.4%	
Number of centers						
Single center	32	66	98	32.7%	24.2-42.4%	0.66
Multicenter	15	33	48	31.2%	19.9-45.3%	
Missing	3	12	15	20.0%	7.0-45.2%	
Enrollment size						
<100	28	49	77	36.4%	26.5-47.5%	0.30
>100	22	60	82	26.8%	18.4-37.3%	
Missing	0	2	2	0.0%	0.0-65.8%	
Recruitment location						
US	14	30	44	31.8%	20.0-46.6%	1.0
International	36	81	117	30.8%	23.1-39.6%	
Blinding						
None	19	15	34	55.9%	39.5-71.1%	0.022
Single	5	18	23	21.7%	9.7-41.9%	
Double	7	26	33	21.2%	10.7-37.8%	
Triple	8	18	26	30.8%	16.5-50.0%	
Quadruple	11	33	44	25.0%	14.6-39.4%	
Missing	0	1	1	0.0%	0.0-79.3%	

Of the 111 completed hip arthroplasty clinical trials, 85 (76.6%) were published, while 26 (23.4%) remained unpublished (Table 2). Among the unpublished studies, 4,262 participants were enrolled in completed trials whose results were not disseminated. Industry-funded trials had a significantly higher nonpublication rate than non-industry-funded trials (39.3% vs. 18.1%, p = 0.022). Intervention type was also significantly associated with publication status (p = 0.044). No statistically significant associations were observed between publication status and the number of centers (p = 0.736), enrollment size (p = 0.311), recruitment region (p = 0.322), or blinding method (p = 0.213).

**Table 2:** Hip arthroplasty clinical trials by publication status.

Characteristic	Published (n = 85)	Unpublished (n = 26)	Total (111)	Nonpublication rate	95% CI	p-value
Funding source						
Non-industry	68	15	83	18.1%	11.3-27.7%	0.022
Industry	17	11	28	39.3%	23.6-57.6%	
Intervention type						
Behavioral	1	0	1	0.0%	0.0-79.3%	0.044
Device	7	4	11	36.4%	15.2-64.6%	
Dietary supplement	1	0	1	0.0%	0.0-79.3%	
Drug	77	17	94	18.1%	11.6-27.1%	
Procedure	10	4	14	28.6%	11.7-54.6%	
Other	5	3	8	37.5%	13.7-69.4%	
Number of centers						
Single center	52	14	66	21.2%	13.1-32.5%	0.736
Multicenter	25	8	33	24.2%	12.8-41.0%	
Missing	8	4	12	33.3%	13.8-60.9%	
Enrollment size						
<100	38	11	49	22.4%	13.0-35.9%	0.311
>100	47	13	60	21.7%	13.1-33.6%	
Missing	0	2	2	100.0%	34.2-100.0%	
Recruitment location						
US	21	9	30	30.0%	16.7-47.9%	0.322
International	64	17	81	21.0%	13.5-31.1%	
Blinding						
None	9	6	15	40.0%	19.8-64.3%	0.213
Single	13	5	18	27.8%	12.5-50.9%	
Double	20	6	26	23.1%	11.0-42.1%	
Triple	15	3	18	16.7%	5.8-39.2%	
Quadruple	28	5	33	15.2%	6.7-30.9%	
Missing	0	1	1	100.0%	20.7-100.0%	

## Discussion

A substantial proportion of registered THA clinical trials were either discontinued (50/161, 31.1%) or remained unpublished following completion (26/111, 23.4%), representing nearly one-third and nearly one-quarter of trials, respectively. Together, these findings indicate that a considerable amount of data generated through THA clinical research may remain unavailable to clinicians, researchers, and patients. Similar patterns have been reported across surgical research, where trial discontinuation and nonpublication remain persistent challenges [3-5].

Trial discontinuation was associated with both intervention type and blinding status. Device trials were more frequently discontinued than drug-based trials. This association may reflect challenges inherent to orthopedic device research, including regulatory requirements, costs associated with device development and implementation, evolving technologies, surgeon learning curves, and difficulties standardizing operative techniques across participating centers [8,9]. These factors may create additional logistical and financial barriers to maintaining enrollment and completing a trial. However, the present analysis cannot determine the specific reasons individual device trials were discontinued, and these findings should therefore be interpreted as associations rather than evidence of causation.

Blinding status was also associated with trial discontinuation, with unblinded trials more frequently discontinued than trials employing greater levels of blinding. One possible explanation is that differences in blinding may reflect broader differences in study infrastructure, available resources, or trial design. Trials capable of implementing more extensive blinding may also have greater methodological or institutional support. However, because this study was observational and relied on registry-level characteristics, it cannot determine whether blinding itself or other characteristics associated with trial design influenced completion. Funding source, enrollment size, number of centers, and recruitment location were not significantly associated with discontinuation, suggesting that the observed differences in completion were not consistently explained by these broader trial characteristics.

Among completed trials, nearly one-quarter remained unpublished. Funding source was associated with publication status, with industry-funded trials demonstrating a greater proportion of nonpublication than non-industry-funded trials. This finding is consistent with previous literature identifying differences in publication patterns according to industry sponsorship [10,11]. Several mechanisms have been proposed to explain industry-associated publication patterns, including selective dissemination based on study findings, sponsor publication priorities, and differences in incentives to publish negative or inconclusive results [10,11]. However, the present study did not evaluate reasons for nonpublication and therefore cannot determine which, if any, of these mechanisms contributed to the observed association.

Publication status also differed according to intervention type, further suggesting that characteristics of the intervention being studied may influence whether completed research ultimately reaches the published literature. In contrast, enrollment size, number of centers, recruitment region, and blinding method were not associated with publication status. Taken together, these findings suggest that nonpublication in THA research is unlikely to be attributable to a single feature of trial design and may instead reflect a combination of investigator, sponsor, journal, and study-specific factors.

The discontinuation and nonpublication observed in this study have important ethical and scientific implications. Individuals who participate in clinical trials do so with the expectation that their involvement will contribute to generalizable medical knowledge and improved patient care [1]. When trials are discontinued without reporting their findings or completed studies remain unpublished, potentially informative data may be unavailable to the broader scientific community. Selective dissemination of study findings can also contribute to publication bias [6,12], potentially distorting estimates of treatment efficacy and safety and influencing clinical decision-making [13].

These concerns are particularly relevant in THA, where implant selection, surgical techniques, and perioperative protocols continue to evolve. Evidence supporting these practices depends on access to both favorable and unfavorable study findings. If negative, inconclusive, or otherwise unpublished findings are systematically absent from the available literature, clinicians, researchers, and guideline developers may be working from an incomplete representation of the evidence [13]. Improved dissemination of findings from completed and discontinued trials may therefore help strengthen the evidence base used to guide future research and clinical practice.

Despite established expectations for trial registration and results reporting, registry information was incomplete for some trials included in this analysis. Incomplete reporting on ClinicalTrials.gov limited our ability to determine the reasons underlying trial discontinuation and, in some cases, to establish whether study findings had been disseminated through other mechanisms [2,14,15]. Greater consistency in updating trial status and reporting results could improve transparency and allow investigators to better understand why orthopedic clinical trials fail to reach completion or publication.

This study has several limitations. The analysis relied primarily on information available through ClinicalTrials.gov, which may be incomplete or outdated. Publication status may also have been misclassified if trial results were published without clear linkage to the corresponding registry record or if identifying information differed between the registry and publication. Trials registered exclusively in international databases, as well as eligible trials that were not publicly registered, may not have been captured. In addition, variability in terminology and registry reporting could have resulted in the exclusion of relevant studies [15]. Finally, because this analysis examined associations between trial characteristics and discontinuation or publication status, the observed relationships should not be interpreted as causal.

Future research should investigate the mechanisms underlying these associations. Direct feedback from investigators through surveys or interviews could help identify logistical, financial, regulatory, and administrative factors contributing to trial discontinuation and nonpublication [16]. Expanding analyses to international trial registries and examining the pathway from trial completion through manuscript submission, peer review, and publication could further clarify where barriers to dissemination occur.

## Conclusions

This study found that nearly one-third of registered THA clinical trials were discontinued and nearly one-quarter of completed trials remained unpublished. Trial discontinuation was associated with intervention type and blinding status, with device trials and unblinded trials demonstrating higher rates of discontinuation. Among completed trials, publication status was associated with funding source and intervention type, with industry-funded trials demonstrating a greater proportion of nonpublication. In contrast, enrollment size, number of centers, and recruitment location were not significantly associated with trial discontinuation or publication status. These findings identify trial characteristics associated with discontinuation and nonpublication and demonstrate that a substantial proportion of registered THA research is discontinued or remains unpublished following completion, limiting the availability of findings within the published evidence base.
